# Oral Pemphigus in Children and Adolescents: A Narrative Review of Published Case Reports

**DOI:** 10.3390/reports9030297

**Published:** 2026-09-02

**Authors:** Filippos Fytros, Panagiota Dimitropoulou, Christina Charisi, Dorothea Pliaka, Nikolaos Spantidakis, Konstantinos Poulopoulos, Maria Fasoula, Efstratios Karagiannidis, Athanasios Poulopoulos, Vasileios Zisis

**Affiliations:** 1 Department of Dentistry, School of Dentistry, European University Cyprus, Nicosia 2404, Cyprus; 2 Department of Oral Medicine and Pathology, School of Dentistry, Aristotle University of Thessaloniki, 54124 Thessaloniki, Greece; 3 Department of Dentoalveolar Surgery, Surgical Implantology and Radiology, School of Dentistry, Aristotle University of Thessaloniki, 54124 Thessaloniki, Greece; 4 Department of Emergency Medicine, Ahepa University Hospital, Aristotle University of Thessaloniki, 54124 Thessaloniki, Greece

**Keywords:** pemphigus vulgaris, pediatric pemphigus, oral manifestations, autoimmune blistering diseases, oral mucosal diseases, diagnosis, treatment

## Abstract

**Background:** Pemphigus is a rare group of autoimmune blistering diseases characterized by autoantibody-mediated loss of keratinocyte adhesion, resulting in intraepithelial blister formation involving the skin and mucous membranes. Pemphigus Vulgaris (PV) is the most common type of pemphigus, which usually presents in adulthood and has a prevalence rate of about 2.83 cases per million person years worldwide. The prevalence rate in children and adolescents is relatively uncommon; however, it accounts for about 1.4 to 3.7 percent of all cases of pemphigus vulgaris reported worldwide. The involvement of oral cavity is clinically significant since it can present prior to the disease or as its predominant feature. Hence, this narrative review seeks to review literature on pemphigus with involvement of oral cavity in children and adolescents. **Objective:** The objective of this study was to review the current literature regarding epidemiology, pathogenesis, clinical presentation, diagnosis, histopathological characteristics, treatment, and outcomes of pemphigus with oral involvement in children and adolescents. **Materials and Methods:** A literature search was conducted using the PubMed/MEDLINE, Scopus, and Cochrane Library databases to identify relevant studies published between 2015 and 2026. The search strategy included terms related to pemphigus, pediatric patients, and oral manifestations. Articles involving patients younger than 18 years of age with oral involvement were screened according to predefined inclusion criteria. Following database screening, duplicate removal, and full-text assessment, 25 studies comprising a total of 51 patients with documented oral or orofacial involvement were included in the final review. **Results:** Pemphigus vulgaris was the predominant subtype, with oral lesions representing the initial or sole manifestation in the majority of patients. The gingiva, mucosa, tongue, lip, and palate were the most common affected areas in the mouth. The lesions in these areas usually appeared as painful erosion, ulcers, and desquamative gingivitis. Histopathology with the presence of acantholysis in the suprabasal area and direct immunofluorescence was the most definitive test for diagnosis. Systemic corticosteroids were the mainstay of treatment in conjunction with steroid sparing agents in some cases, with good results in difficult cases using rituximab. Overall, most patients achieved partial or complete clinical remission following appropriate treatment, although relapses were occasionally reported. **Conclusions:** Pemphigus in children and adolescents is rarely encountered; however, it should be included as a differential diagnosis of erosive/ulcerative lesions. Early identification, diagnosis, and treatment through a collaborative approach from dental practitioners are critical. Further research is required at multiple centers to gain a better understanding of the disease and to develop evidence-based guidelines for diagnosis and treatment of pediatric patients.

## 1. Introduction

The lesions that affect oral mucosa are commonly found in children and include a diverse range of infections, inflammations, trauma, developmental disorders, and autoimmune processes. Despite the majority of oral lesions found in children being benign, it is possible that in rare cases the mouth can be the primary site of a systemic or immune-related disorder. Therefore, any erosive or ulcerative lesion that is persistent, recurrent, or unusual should be carefully examined, especially if it hinders oral functions or cannot be associated with the common childhood problems [1,2].

Pemphigus is a collection of rare and autoimmune diseases associated with immune-mediated mucocutaneous diseases where the immune system attacks the desmosome proteins of keratinocytes leading to blistering of the skin and/or mucous membrane because of the lack of cellular adhesion. The most common and clinically significant subtype of pemphigus is pemphigus vulgaris (PV), which is caused by the presence of autoantibodies targeting desmoglein 3 (Dsg3), and, in case of mucocutaneous disease, desmoglein 1 (Dsg1). Disruption of desmosomal cellular adhesion leads to acantholysis and formation of fragile vesicles/bullae, which easily break down, causing painful erosion, especially on the mucous membranes [3,4].

There is special clinical significance of the oral cavity in PV. Sometimes, the lesions in the mouth precede the skin lesions, and sometimes in the mouth, the only lesion or predominant manifestation of the disease may be noted. Since the blisters within the mouth are rarely intact, PV appears as erosions or ulcers in the oral mucosa, tongue, palate, gingiva, and labial/buccal mucosa. Involvement of the gingiva may occur as desquamative gingivitis or in some cases atypical gingival lesions. Such oral manifestations affect the process of mastication and deglutition and, in case of their severity and long duration, may affect oral intake and nutrition [5,6,7].

Pemphigus typically occurs among adults and the occurrence in children and adolescents is uncommon. Juvenile or pediatric pemphigus vulgaris (PV) as well as PV of adolescents only constitutes a minor number of all PV cases that have been documented. However, due to its rarity, one should not overlook the diagnosis of PV among children presenting with erosive and vesiculobullous conditions in the mouth. Though the disease is similar to adult disease in terms of its presentation, histology, and immunology, rare incidence and association with common pediatric diseases in the mouth can greatly affect its diagnosis [7,8].

Such a diagnostic dilemma becomes especially relevant if the lesions in the mouth are the first and only presenting symptoms of the condition. Childhood PV may present similar to recurrent aphthous stomatitis, viral or other types of infectious stomatitis, inflammatory erosive conditions, and other autoimmune diseases involving blisters formation. Thus, diagnosis requires a correlation of the clinical picture with pathological changes found on histological and immunopathological examination. The characteristic feature of PV histopathology is suprabasal acantholysis, while direct immunofluorescence usually shows the presence of IgG deposited between cells in the epidermis, sometimes in combination with complement. Serological testing for the presence of autoantibodies directed against desmoglein 3 and desmoglein 1 provides complementary diagnostic and monitoring data [4,8,9].

The early diagnosis is of utmost importance in children because the ongoing painful lesions in the mouth affect eating and swallowing and can result in weight loss and malnutrition. In addition, the treatment of pemphigus in children and adolescents poses several problems because one should manage to reach appropriate control without causing harm from prolonged systemic use of corticosteroids and immunosuppression in growing children. While the therapy was traditionally focused on the use of steroids and steroid-sparing immunosuppression, there is an increased utilization of biologics, especially rituximab, in case of severe and resistant forms of pemphigus. However, data specifically related to the treatment of children and adolescents is limited, and the protocols for children have yet to be developed [7].

Although pemphigus in children and adolescents has been previously reviewed, the available literature has primarily focused on its dermatological characteristics, with limited emphasis on oral manifestations. Given that oral lesions may represent the initial or, in some cases, the only manifestation of the disease, a focused evaluation from an oral medicine and oral pathology perspective is clinically relevant. Therefore, the present narrative review aims to synthesize the available evidence on pemphigus in children and adolescents with documented oral involvement, with particular emphasis on oral clinical presentation, diagnostic and histopathological features, treatment approaches, clinical outcomes, associated systemic conditions, and potential triggering factors.

## 2. Materials and Methods

This study was conducted as a structured narrative review evaluating the oral manifestations, diagnostic features, treatment approaches, and clinical outcomes of pemphigus in children and adolescents.

A comprehensive literature search was conducted in July 2026 in the PubMed/MEDLINE, Scopus, and Cochrane Library databases to identify relevant studies published between 2015 and 2026. The search strategy was initially developed for PubMed using free-text keywords searched within the title and abstract fields and was subsequently adapted for Scopus and the Cochrane Library according to the syntax requirements of each database.

The PubMed search strategy was constructed as follows:

(“pemphigus vulgaris”[Title/Abstract] OR “juvenile pemphigus”[Title/Abstract] OR “pediatric pemphigus”[Title/Abstract] OR “childhood pemphigus”[Title/Abstract] OR “paraneoplastic pemphigus”[Title/Abstract]) AND (oral[Title/Abstract] OR “oral mucosa”[Title/Abstract] OR “oral lesions”[Title/Abstract] OR gingiva*[Title/Abstract]) AND (child*[Title/Abstract] OR pediatric*[Title/Abstract] OR paediatric*[Title/Abstract] OR adolescen*[Title/Abstract] OR juvenile[Title/Abstract]).

The search strategy was adapted to the syntax and indexing requirements of each database while maintaining the same core concepts related to disease, oral involvement, and the population of children and adolescents. The PubMed search, restricted to publications from 2015 to 2026, yielded 30 records. Following title and abstract screening and full-text assessment, 18 studies fulfilled the eligibility criteria. Searches of Scopus and the Cochrane Library identified eight additional eligible studies after duplicate removal, resulting in a total of 25 studies included in the final review.

Studies were eligible if they were published between 2015 and 2026, involved patients younger than 18 years with a confirmed diagnosis of pemphigus, and reported oral or orofacial involvement. Case reports, case series, and observational studies providing original clinical data were included. Patients with concomitant systemic diseases, syndromic conditions, or other comorbidities were not excluded, provided that they fulfilled the aforementioned eligibility criteria and had a confirmed diagnosis of pemphigus with oral or orofacial involvement. Studies were excluded if they involved exclusively adult populations, reported autoimmune blistering diseases other than pemphigus, lacked oral or orofacial involvement, or provided insufficient clinical information. Reviews, editorials, conference abstracts, and publications without original patient data were also excluded.

Data extracted from each study included the author and year of publication, patient age and sex, location and clinical presentation of oral lesions, histopathological and immunopathological findings, treatment modalities, and clinical outcomes. Due to the heterogeneity of the available evidence, the findings were synthesized descriptively and summarized in tabular form.

## 3. Results

Following database screening, duplicate removal, and full-text assessment, 25 studies comprising 51 patients with documented oral or orofacial involvement were included in the final review. The characteristics of the included studies and patients are presented in Table 1, while the main demographic and clinical findings, treatment approaches, and outcomes are summarized in Table 2.

### Treatment and Clinical Outcomes

There were great variations among treatment methods depending on the severity of the disease, the involvement of mucosa and skin, and the type of pemphigus. Nevertheless, systemic corticosteroids constituted the cornerstone of therapy in the vast majority of patients. The drug could be in form of oral or intravenous prednisolone, methylprednisolone, or dexamethasone either alone or more commonly in combination with other immunosuppressants that could replace steroids. Azathioprine and mycophenolate mofetil were the commonest of such drugs, especially for persistent disease, relapse, and widespread mucocutaneous involvement. Dapsone was also used in some patients, though in one case, the drug had to be stopped owing to side effects [8]. On the other hand, in those patients with only oral disease, topical steroids could be effective without requiring systemic immunosuppression [5,7,8,10,11,12,13,15,17,19,21,22,24,25,26,29,30,31].

Overall, patients with localized oral disease generally required less intensive therapeutic regimens than those presenting with extensive mucocutaneous or multisystem involvement, who more frequently received combination immunosuppressive therapy and biologic agents [5,7,17,18].

In general, almost all patients showed good response to the treatment. Improvement in painful oral erosions was noted two to five weeks after the start of treatment, allowing for normal oral intake and healing of the lesions. Nutritional improvement, weight gain, and improvement in quality of life were also reported among others due to disease control, especially in children with significant feeding problems and malnutrition [11,12,13,19,24,25,29,30].

Despite this initial favorable response, however, total remission was not always attained by first-line therapy. Recurrence of the disease was quite common among all the cases discussed above. Some of the patients relapsed either when tapering off corticosteroids or soon after discontinuing therapy, necessitating the reinitiation of corticosteroids or the intensification of immunosuppressant therapy. In the same way, the large retrospective study of children revealed that even though all of the patients had complete remission of the disease while on medication, more than half of them relapsed in the course of follow-up. Oral lesions took longer to go into remission compared to cutaneous lesions [8,11,15,17,21,31].

In almost all such patients, use of rituximab produced significant improvement in symptoms or complete disease remission, enabling tapering or cessation of systemic corticosteroids [10,14,17,18,21,23,28,31]. However, in some rare instances, there was an incomplete response, necessitating resorting to other methods of treatment such as rituximab desensitization [20,26].

Data concerning the effectiveness of novel biologic methods for pediatric pemphigus are scarce. Dupilumab was used in one case with refractory disease affecting the oral cavity, skin, and esophagus with long-lasting remission being achieved [21]. In addition, desensitization using rituximab in the presence of rituximab allergy reaction occurred in one other case [25]. These two cases cannot draw definite conclusions about the effectiveness of these treatments.

Paraneoplastic pemphigus patients had significantly divergent treatment needs and prognoses. In almost all these patients, treatment involved surgical removal of the concomitant Castleman disease in addition to heavy immunosuppressive therapy. Surgical removal of the causative tumor usually led to significant improvement or regression of the mucocutaneous disease. Nevertheless, although there was successful control of the oral disease, some patients developed bronchiolitis obliterans that resulted in respiratory failure, need for ventilation, lung transplant, or even death. Conversely, pemphigus vulgaris in children seldom led to serious systemic complications [14,16,18,20,23,27,28].

Supportive management was also considered an important aspect of the therapy. Nutritional rehabilitation, diet changes, infection prevention, wound care, oral hygiene, use of topical steroids, and treatment of secondary infections often became a part of the treatment regimen [7,12,21,29,30].

## 4. Discussion

The aim of this study was to review the most recently published literature on pemphigus with oral mucosal involvement in children and adolescents, paying special attention to epidemiological features of such patients and accompanying diseases, clinical manifestations, diagnosis, methods of treatment used and the outcomes. The topic of pemphigus vulgaris in children and adolescents is not entirely new one. The current review is based on results of the previously conducted reviews [7,32], its novelty however relies in its specific oral medicine and oral-pathology focus, including only cases with oral mucosa involvement, while encompassing a broader pediatric age range (0–18 years). Furthermore, the present review uniquely reports on potential associated comorbidities, medical history, and potential triggering factors [6,7].

Overall, the key results obtained from this literature review show that oral erosions and ulcers are among the typical signs of pediatric pemphigus and can be the first or even the only sign of the disease [5,10,17]. The involvement of the skin, eye and genitals was also mentioned [10,11,17], whereas the diagnosis depended on clinical evaluation supplemented with histopathology, direct immunofluorescence and serology testing [10,11,17].

In the present review, oral involvement represented the predominant clinical presentation, as only studies reporting oral manifestations were included. While only a minority of patients had only lesions involving their mouths, the majority of patients had lesions involving both oral cavity and skin, with the skin, eyes, and genital mucosa being the most common areas affected other than the mouth. These findings are consistent with previously published evidence, in which 16.2% (*n* = 6/37) of patients had isolated oral lesions, while 78.4% (*n* = 29/37) had mucocutaneous disease involvement [7]. PV in children and adolescents has similar presentation to the adult PV; it involves blistering condition in which there are very fragile vesicles and bullae which break easily causing painful erosion. Ocular and genital involvement has also been reported among children with PV [7]. Also, the current review revealed that oral lesions cause sialorrhea, dysphonia, trismus, and difficulty in oral intake, which may cause malnutrition in the case of prolonged feeding problems in the pediatric patient [8,22,29].

All in all, childhood PV is seen to have the same main features as PV in adults, though there are certain aspects that are particularly important in children. First, oral manifestation can affect feeding and swallowing and, therefore, lead to such complications as growth retardation due to nutrition problems. Also, in childhood PV ocular and genital manifestations have been noted and seem to be significant from the point of view of clinical symptoms of the disease [11,12].

Despite mucocutaneous manifestations being more common generally, a number of the patients had isolated oral lesions throughout their disease course, emphasizing the fact that isolated oral lesions could be the first—and sometimes only—sign of juvenile PV. Oral lesions included gingival involvement, desquamative gingivitis, oral ulcers, erosion of buccal mucosa and tongue, along with painful lesions of the lips [5,7,13,25].

In addition, the current review also emphasizes the large variability in the severity of the oral disease. For example, while some of the children had only localized gingival erosions that were well-controlled with just topical steroids, other children suffered from widespread oral ulcers affecting almost the whole mouth and causing severe pain, difficulty swallowing, drooling, poor nutrition, and significant weight loss [5,19,22,29].

Impairment of feeding was another clinical consequence which was among the most significant of those related to oral manifestations. There have been various descriptions of decreased oral intake, malnutrition, failure to thrive, or delay in weight gain as a result of pain caused by oral erosion, underscoring the importance of nutritional management as part of treatment [12,22,29,30].

The diagnosis of pemphigus vulgaris is founded upon the analysis of clinical, histopathological, and immunological data, as suggested by the 2015 S2 European Dermatology Forum Guidelines [4]. The diagnostic process starts with the collection of patient’s history, including family history and possible triggers, as well as clinical examination that involves evaluation of lesion characteristics, detection of the Nikolsky sign, and overall condition of the patient [4].

The results obtained during this review confirm current diagnostic guidelines since diagnosis of all patients under discussion was made using a complex of clinical, histopathological, direct immunofluorescent tests, and serology. Histopathological features included the presence of suprabasal acantholysis without involvement of the basal keratinocyte layer, which causes the characteristic appearance of “row of tombstones”, and acantholytic (Tzanck) cells in combination with inflammatory cells infiltrate in the dermal connective tissue. Perilesional skin direct immunofluorescent tests revealed intercellular deposits of IgG and C3, while indirect immunofluorescence and ELISA revealed circulating autoantibodies against desmoglein (DSG) 3 and/or DSG1 [33,34,35]. As expected according to existing data, high titers of anti-DSG3 autoantibodies were revealed in the majority of cases of pemphigus vulgaris, and anti-DSG1 autoantibody titer was different [10,11,17,19,20]. Higher titers of autoantibodies were correlated with increased activity of disease and could indicate disease relapse [25].

Nevertheless, several reports demonstrated that diagnosis may occasionally be challenging. Initial direct immunofluorescence was negative in some patients despite compatible clinical and histopathological findings, whereas uncommon antibody profiles, including isolated anti-desmocollin 3 antibodies, were described in rare variants of juvenile pemphigus. These observations emphasize that a negative individual diagnostic test should not exclude the disease when clinical suspicion remains high [19,31].

Furthermore, repeated biopsies or additional immunological investigations were required in several complex cases before a definitive diagnosis could be established, particularly in patients with paraneoplastic disease or atypical clinical presentations [14,20,23,28].

In most cases, the rare occurrence of pemphigus vulgaris in children and adolescents is responsible for a great number of difficulties in making an appropriate diagnosis, causing late detection and delayed treatment [7]. In fact, oral lesions need to be differentiated from a range of diseases with quite similar presentation, including erosive oral lichen planus, Behçet’s disease, mucous membrane pemphigoid, recurrent aphthous stomatitis, and infections with viruses like herpes simplex virus (HSV), which are commonly diagnosed among children. Moreover, epidermolysis bullosa, a group of genetically determined blistering skin diseases known as “genetic pemphigus”, has to be separated from autoimmune types of pemphigus, such as family- and endemic pemphigus [36,37]. In some cases, the wide range of clinical presentations might cause even greater difficulties during differential diagnosis. For example, a previous study described a patient with diffuse gingival hypertrophy, which is probably caused by mucosal edema, being the sole feature of pemphigus vulgaris [31]. Similar cases are known from literature and illustrate that isolated gingival enlargement needs to be differentiated from the other common childhood disease, such as hereditary or medication-associated gingival fibromatosis, prior to diagnosing PV [7,31].

The present review also highlights that prolonged diagnostic delay remains a common problem. Several children were initially diagnosed with more prevalent conditions, including aphthous stomatitis, Behçet disease, bullous impetigo, infectious stomatitis, or lichen sclerosus, before the correct diagnosis of pemphigus was established. Such delays frequently resulted in disease progression, nutritional deterioration, or the development of extensive mucocutaneous involvement [8,15,23,38].

Importantly, isolated gingival disease may represent the only manifestation of PV. Desquamative gingivitis, diffuse gingival hyperplasia, or gingival erosions in an isolated form are additional evidence of the crucial role that dentists play in diagnosing these rare cases and performing biopsy [5,13,31].

Among the autoimmune blistering disorders affecting children, mucous membrane pemphigoid (MMP) represents an important differential diagnosis of pemphigus vulgaris, although it is considerably less common in the pediatric population [38]. In both these cases, the presentation can include painful erosions of mouth, desquamative gingivitis and positive Nikolsky sign, hence making the clinical distinction between the two challenging in the initial phases of the illness. However, there is a fundamental difference in the pathogenesis and histological features in these two cases, with MMP being a subepithelial blister disease having linear deposition of immunoreactants in the basement membrane zone, and PV being a suprabasal acantholytic disease with intracellular IgG deposits in the epithelium. This difference is vital since diagnosis depends on histology and immunofluorescence studies [38].

Pediatric pemphigus vulgaris is extremely rare with most cases occurring in adulthood despite a family history of the disease [4,33]. However, there exist two distinct exceptions to the above-mentioned trend. The first is endemic pemphigus foliaceus (PF), which is commonly seen in children and young adults residing in endemic areas, where a combination of genetic predisposition and environmental factors like recurrent bites by hematophagous insects including Lutzomyia longipalpis has been shown to play a role in disease development [34]. The second exception is that of neonatal pemphigus vulgaris. This is a temporary state associated with children born to mothers suffering from pemphigus vulgaris. Here, the transfer of maternal autoantibodies through the placenta leads to temporary development of bullous and erosive skin lesions on the child, which heal after removal of the maternal autoantibodies from the system of the child [35,36].

Pediatric pemphigus vulgaris makes up only 1.4–3.7% of all the cases of PV, which highlights the rare occurrence of this disorder among children [7]. Usually, PV is diagnosed in adolescence, when the age of the first symptoms’ appearance is about 12 years old, and its incidence increases with age progression [7,35]. Although PV is generally more common in females [37], the present review and recent pediatric evidence indicate a male predominance, with a male-to-female ratio of approximately 2.33:1 [7]. This finding can probably be explained by the small number of cases in children because there are no other hypotheses suggested in the scientific literature on this problem. However, even in such a rare disease, the data presented in the current review shows that adolescents are the period of the highest probability of disease onset, while pre-five age cases are uncommon and more extensive but still possible. Nevertheless, the clinicians have to remember that young age does not rule out the diagnosis since some cases of PV were diagnosed in early childhood [15,22,29,30]. Also, it was possible to identify several other related diseases during the current review, such as eosinophilic esophagitis, protein-losing gastroenteropathy, and viral infections. Eosinophilic esophagitis is not very frequent but has been known to occur in patients with PV, and the presence of these two conditions together may imply certain similarities in their pathophysiology [11,39]. The same applies to protein-losing gastroenteropathy, which can be one of the complications of PV because of the damage that it causes to the mucosa [30,40]. Viral infection, especially varicella-zoster virus (VZV), may be linked to the development of pemphigus [41,42].

The present review also demonstrated that pediatric pemphigus encompasses a broader clinical spectrum than previously appreciated. While the most common type is PV, there are rare forms such as pemphigus vegetans and paraneoplastic pemphigus (PNP), which is also known as paraneoplastic autoimmune multiorgan syndrome (PAMS). Even though these are extremely rare conditions, one should bear them in mind while making a differential diagnosis for cases of persistent ulcers in the mouth, especially with severe systemic features [12,18,20,23].

Another noteworthy observation of the present review was the relatively frequent coexistence of pemphigus with systemic disorders. Aside from the eosinophilic esophagitis and the protein-losing gastroenteropathy, there are a number of new cases which have shown a close connection between PNP and Castleman disease. Painful oral erosions were found to be an early symptom of the malignancy [16,18,20,23,28].

Such observations further validate the notion that oral ulcers occurring in children should not always be considered to be an isolated condition of the mouth. On the contrary, clinicians should have a high level of suspicion for the presence of an underlying systemic disorder, particularly if treatment is ineffective or other symptoms occur [18,20,23,27].

Interestingly, respiratory involvement was observed almost exclusively in patients with PNP/PAMS rather than classical PV. Progressive obliterative bronchiolitis appeared to be the most serious extragastrointestinal complication, contributing greatly to morbidity and mortality despite rigorous immunosuppressive therapy. Such observation underscores the distinct pathogenesis of PNP from classic PV [14,18,23,27].

At present, the view on the pathogenesis of pemphigus is much more advanced than the classical paradigm according to which acantholysis occurs due to the interaction of autoantibodies with desmosomal cadherins. Recent research proves the existence of the complex network of signaling cascades, including p38-MAPK and ERK, whose activation leads to the disruption of keratinocyte intercellular contact and their death. Also, the involvement of innate and adaptive immunity mechanisms in the development of the disease attracts attention. Apart from the critical importance of the B- and T- cellular response in the development of disease and maintenance of inflammation, such phenomena as pyroptosis and disruption of neonatal Fc receptor (FcRn) have been proven to be essential factors in the pathogenesis of pemphigus [43,44].

Despite the progress in unraveling the mechanism of pemphigus, systemic corticosteroids continue to be the mainstay of treatment. Standard immunosuppressive treatments, such as azathioprine and mycophenolate mofetil, are often used either as adjuvant medications or in conjunction with systemic corticosteroids to enhance efficacy [43]. While the pediatric PV was always believed to be more favorable than the adult disease from the prognostic point of view, treatment results still vary widely between patients [7]. The examples of such problems can be found in several cases analyzed in the current paper. In particular, due to the adverse effects both locally and systemically associated with long-term use of corticosteroids, especially in young patients, there is always a need for dose tapering. This might become one of the reasons of relapse [8,15,22]. In addition, immunosuppressants could be discontinued because of the adverse effects of the treatment [7]. Due to the rarity of pediatric PV, no standard treatment regimen has been developed so far [7]. However, the results of the current review, along with those of Sriram et al., confirm the increasing role of biological therapy in treating refractory disease. Although the majority of patients were responsive to systemic corticosteroids in combination with immunosuppressant drugs, a few resistant cases eventually went into remission after being treated with rituximab, while more recent research has confirmed positive results from dupilumab and rituximab desensitization in some selected patients who failed to respond to earlier therapies [7,10,21,22,26]. Lastly, proper management of the disease involves not only drug therapy but also overall supportive treatment, such as proper nutrition, preventive dentistry, infection prevention, and regular medical check-ups [43]. More extensive case series involving children have demonstrated that, while achieving remission in most patients is possible, oral lesions heal slower than skin lesions, and relapse happens relatively frequently [17].

While there have been good results seen with corticosteroids, immunosuppressants, and rituximab; however, these should be carefully taken into consideration since the majority of evidence for all of this is based on case reports, small case series and retrospective studies, with varying protocols in treatments and their evaluations. Consequently, no clear conclusion can be drawn about which therapy approach may be more effective.

### Limitations

The results of this literature review need to be considered in the context of a number of limitations. Firstly, the fact that the prevalence of pediatric pemphigus vulgaris is relatively low and only a few studies have been carried out, with the majority being case reports and series, limits the applicability of the results obtained and raises concerns about selection and publication bias. Also, inconsistency in the nomenclature of the efficacy of treatment, its duration, and the clinical response does not allow any statistical comparison between different studies. Furthermore, follow-up duration was variable and incompletely reported in several studies, limiting the assessment of long-term treatment outcomes and disease recurrence. Thus, future studies should be focused on the development of collaborative multicenter prospective research projects aimed at the creation of unified diagnostic and treatment guidelines for pediatric PV, the identification of specific genetic and environmental predisposing factors, and evaluation of targeted therapy, especially monoclonal antibodies.

## 5. Conclusions

The clinical characteristics of pemphigus vulgaris in children and adolescents appear largely similar to those observed in adults, although ocular and genital involvement may be more frequently reported. Oral lesions may cause significant functional impairment, including difficulty with eating, and in severe cases may contribute to malnutrition and impaired development. Early recognition, particularly when oral lesions represent the initial manifestation, and multidisciplinary management are therefore important. However, pemphigus vulgaris is rare in children and adolescents, and the available evidence is limited and largely derived from case reports and small case series. Consequently, conclusions regarding treatment effectiveness and recommendations for specific therapeutic approaches should be interpreted with caution, and further well-designed studies are needed to establish more robust evidence for the management of this population.

## Figures and Tables

**Table 1:** The characteristics of the included studies and patients.

Study	Age/Sex	Site	Clinical Presentation	Histopathological Findings
Chen et al., 2016 [10]	17/M	Oral mucosa: outer lips, hard and soft palate; subsequently skin of neck, hands, trunk, extremities, groin and buttocks	Three-month history of severe painful and persistent oral ulcers with crusting and necrotic tissue. Subsequently developed multiple vesicles and bullae, including relatively tense serous bullae, disseminated over the body	Histopathology showed suprabasal acantholysis. Direct immunofluorescence demonstrated intercellular IgG and C3 deposition, predominantly in the suprabasal epidermis
Gue et al. (2017) [11]	13/M	Oropharynx, buccal mucosa, gingiva, lateral borders of the tongue, lips, and oral commissures	Five-week history of severe, widespread oral vesiculobullous lesions and ulcerations associated with lip swelling, transient anal ulcerations, conjunctival congestion, significant weight loss, and eosinophilic esophagitis	Extensive suprabasal epithelial acantholysis with loss of the superficial epithelial layers and a predominantly plasma-cell inflammatory infiltrate. DIF demonstrated intercellular epithelial IgG staining with C3 deposition along the mucosal–lamina propria junction. Anti-DSG3 antibodies were strongly positive (>1:2560)
Suwarsa et al. (2017) [12]	12/M	Oral and perioral regions; axillae, trunk, extremities, and perianal region	Severe oral pain and stomatitis resulting in impaired oral intake, followed by widespread clear fluid-filled blisters, erosions, hemorrhagic crusts, and hypertrophic verrucous vegetative plaques. Severe malnutrition (marasmus) was also present.	Suprabasal cleft with scattered acantholytic cells, hyperkeratosis, and subepithelial lymphocytic infiltration. DIF revealed intercellular IgG deposits on the surface of keratinocytes, supporting the diagnosis of pemphigus vegetans.
Bajpai et al. (2016) [13]	11/F	Anterior gingiva, soft palate, and buccal mucosa	Three-month history of chronic oral ulcerations affecting normal oral function. Multiple erosive and erythematous oral lesions were observed. No cutaneous lesions were present.	Tzanck smear revealed rounded acantholytic keratinocytes with enlarged nuclei and perinuclear halos. Histopathology demonstrated marked suprabasal epithelial splitting, with a single basal cell layer remaining attached to the basement membrane and numerous acantholytic cells. DIF showed intercellular IgG deposition.
Han et al. (2019) [14]	13/F	Tongue and buccal mucosa; scalp, face, and hands	Recurrent painful oral ulcers for 6 months, followed by violaceous lichen planus-like lesions. The patient subsequently developed bronchiolitis obliterans and progressive respiratory failure associated with unicentric Castleman disease.	Oral biopsy showed suprabasal acantholysis with submucosal lymphoplasmacytic infiltration. Skin biopsy revealed lichenoid interface dermatitis, keratinocyte necrosis, vacuolar interface changes, and band-like lymphoplasmacytic infiltration. DIF was negative, while IIF demonstrated circulating anti-intercellular substance antibodies.
Casuriaga Lamboglia et al. (2018) [15]	2/M	Oral mucosa and lips; skin lesions involving the umbilical region, face, trunk, upper and lower limbs, and genital region; anal mucosal involvement	Initially presented with generalized cutaneous blisters without mucosal involvement and was misdiagnosed with bullous impetigo. Subsequently developed multiple painful oral erosions, crusted lip lesions, feeding difficulty, dysphonia, extensive anal erosions, and a positive Nikolsky sign.	Histopathology demonstrated a suprabasal intraepidermal blister with acantholytic cells and basal keratinocytes remaining attached to the basement membrane (“row of tombstones”), accompanied by lymphomononuclear inflammatory infiltrates and eosinophils. DIF showed intercellular intraepidermal deposition of IgG and C3.
Le et al. (2019) [5]	14/F	Marginal gingiva	Nine-month history of localized gingival ulcerations. Clinical examination revealed mild erosions along the marginal gingiva and a positive Nikolsky sign. No cutaneous or other mucosal involvement was observed.	Diagnosis of oral PV was confirmed by histopathological examination and DIF. ELISA demonstrated strongly positive anti-DSG3 antibodies (109.79 U; normal <9.0) and negative anti-DSG1 antibodies.
Lins Filho et al. (2021) [8]	8/M	Upper and lower labial mucosa, ventral tongue, hard and soft palate, oral commissures, lower alveolar mucosa, and upper and lower gingiva; pharynx, larynx, lower eyelid, and genital mucosa	Recurrent oral ulcerations beginning at 3 years of age, initially affecting the tongue and subsequently spreading throughout the oral cavity. At presentation, extensive painless oral ulcers were associated with limited mouth opening, sialorrhea, speech difficulty, and hoarseness. No cutaneous involvement occurred.	Intraepithelial blister with suprabasal clefting, acantholytic cells, and retention of basal keratinocytes attached to the connective tissue. A lymphoplasmacytic inflammatory infiltrate with eosinophils was present. DIF demonstrated intercellular IgG3 deposition in the remaining basal epithelium.
Sathishkumar et al. (2021) [16]	8/F	Oral, genital, and ocular mucosa; skin	Persistent, painful pemphigus-like oral mucositis associated with lichen planus-like cutaneous lesions. Paraneoplastic autoimmune multiorgan syndrome was associated with retroperitoneal Castleman disease.	Intraepidermal acantholysis, interface dermatitis with thickening of the basement membrane, and subepidermal clefting. DIF demonstrated granular basement-membrane deposition of IgG, IgA, IgM, and C3.
Sathishkumar et al. (2021) [16]	8/M	Oral and ocular mucosa; skin	Persistent, painful pemphigus-like oral mucositis associated with lichen planus-like lesions and a generalized erythematous papular eruption. Paraneoplastic autoimmune multiorgan syndrome was associated with cervical Castleman disease.	Intraepidermal acantholysis, interface dermatitis, apoptotic keratinocytes, and subepidermal clefting. DIF demonstrated linear basement-membrane deposition of IgG, IgM, and C3.
Mahajan et al. (2022)—Retrospective case series [17]	24 patients; mean age at onset: 14.7 years; M/F: 15/9	Oral mucosa (23/24); genital mucosa (5/24); nasal mucosa (2/24); skin in patients with mucocutaneous disease	Oral involvement was present in 23/24 patients and represented the initial disease site in 21/24. Eighteen patients had mucocutaneous PV, five had mucosa-limited disease, and one had cutaneous-limited PV. Oral lesions were more treatment-refractory than cutaneous lesions.	Diagnosis was established based on clinical, histopathological, and direct immunofluorescence findings; individual microscopic and immunopathological findings were not reported.
Barry et al. (2023)—Multicenter case series [18]	4 patients aged <18 years; 7–12 years; 2 M/2 F	Oral mucosa (4/4); ocular mucosa (2/4); anogenital mucosa (2/4); skin	All patients had persistent oral mucositis associated with heterogeneous cutaneous manifestations. All had Castleman disease; three had unicentric and one had multicentric disease. Three developed bronchiolitis obliterans.	IIF on monkey esophagus was positive in all patients and IIF on rat bladder was positive in all four. Anti-Dsg3 antibodies were detected in all patients, while anti-Dsg1 antibodies were detected in two.
Vezzoli et al. (2022) [19]	9/M	Upper and lower lips, gingiva, tongue, hard and soft palate, bilateral buccal mucosa; later skin involvement	Extensive, painful, symmetrically distributed oral erosions, initially accompanied by low-grade fever, malaise, and bilateral conjunctivitis. Six months later, worsening oral lesions with halitosis, hoarseness, and moderate dysphagia, along with hyperpigmented lesions on the back.	Oral biopsy showed acantholysis with an intact basal layer; initial DIF was negative. Subsequent skin biopsy demonstrated intercellular IgG and C3 deposits in a fishnet pattern. ELISA showed high anti-Dsg3 antibody levels and negative anti-Dsg1 antibodies.
Irrera et al. (2023) [20]	13/F	Oral mucosa, gingiva, tongue, lips; conjunctiva; skin	Recurrent painful erosive oral mucositis for 2 years, with tongue pyogenic granuloma-like lesions. Later, severe gingivitis, tongue erosions, hemorrhagic lip crusts, conjunctival hyperemia, and polymorphic vesiculobullous skin lesions developed.	Epidermal hyperplasia, spongiosis, and upper dermal lymphocytic infiltrate. DIF: intercellular IgG/C3 throughout the epidermis; IIF: epithelial cell-surface IgG. Elevated anti-Dsg1/3 antibodies.
Akham et al. (2023) [21]	Preteen/M	Hard palate, bilateral buccal mucosa; skin	Multiple jagged oral erosions with rapidly progressive, extensive flaccid bullae and cutaneous erosions (30–60% BSA); complicated by *K. pneumoniae* sepsis.	Suprabasal clefting with acantholytic cells; Tzanck smear showed numerous acantholytic cells. Histopathology and DIF were consistent with PV.
Sriram et al. (2024) [6]	16/F	Bilateral buccal mucosa; lateral and ventral tongue	Oral soreness and burning for 8 months, with painful diffuse irregular erosions, pseudomembranous slough, difficulty chewing and swallowing, and positive Nikolsky sign; no cutaneous or other mucosal involvement.	Suprabasal epithelial split with acantholysis and “tombstone” basal cells. DIF showed intercellular IgG in a fishnet pattern; elevated anti-Dsg1 and anti-Dsg3 antibodies.
Yun et al. (2025) [22]	3/M	Lips, oral mucosa, gingiva, esophagus; later skin and nails	Painful irregular oral erosions with white pseudomembranes, food aversion, failure to thrive, and esophageal involvement. Later developed cutaneous blistering and onychomadesis.	Esophageal biopsy showed acantholysis, suprabasal clefting, and marked intraepithelial eosinophilia. DIF demonstrated strong intercellular IgG/IgG4 epithelial reactivity; markedly elevated anti-Dsg3 antibodies.
Zhang et al. (2024) [23]	10/F	Lips, tongue, oral mucosa, conjunctiva, genital mucosa; later skin, nails, and hair	Recurrent painful oral ulcers lasting >3 months that enlarged and coalesced, with swollen fissured lips, hemorrhagic crusts, ulcerated tongue, limited mouth opening and tongue movement, and impaired eating. Initially misdiagnosed as Behçet disease; later developed conjunctivitis, generalized rash, blisters, alopecia, and nail involvement.	No oral or skin biopsy was performed. Histopathology of the mediastinal mass showed lymphoid hyperplasia consistent with hyaline-vascular unicentric Castleman disease.
Belgaumkar et al. (2025) [24]	7/F	Buccal mucosa, inner lip; face, scalp, trunk, limbs, vulval and perianal regions	Few oral erosions with intolerance to spicy food, accompanied by widespread vesicles, flaccid bullae, crusted erosions, and positive Nikolsky sign.	Suprabasal cleft with basal “tombstone” appearance and acantholytic cells on Tzanck smear; DIF showed intraepidermal fishnet-pattern IgG deposition.
Govindarajulu et al. (2025) [25]	12/M	Bilateral buccal mucosa, tongue; glans penis	Burning oral sensation with progressive, painful erythematous erosions of the buccal and lingual mucosa, aggravated by spicy foods. Deep dorsal and lateral tongue fissures with whitish coating produced a characteristic scrotal tongue appearance; genital erosions and fissures were also present.	Tzanck smear showed acantholytic cells. Tongue biopsy revealed suprabasal clefting, basal “tombstone” appearance, few acantholytic cells, and dense subepithelial lymphoplasmacytic infiltration with interstitial hemorrhage. DIF showed intercellular IgG deposition.
De Felici Del Giudice et al. (2023) [26]	12/M	Gingiva, oral mucosa, hard palate, pharynx; conjunctiva and skin	Severe gingivostomatitis with painful oral erosions, extensive gingival de-epithelialization, hard-palate and pharyngeal lesions, dysphagia, and subsequent widespread cutaneous bullae.	Suprabasal acantholysis on histopathology; DIF showed intercellular IgG deposition. ELISA detected high anti-Dsg3 IgG levels and BP180 antibodies.
Fidder et al., 2021 [27]	7-year-old female	Lips, tongue, buccal mucosa, and esophagus	Severe painful stomatitis; erosive cheilitis; oral ulcerations and blisters; painful erythematous tongue lesions; white plaques and lichenoid lesions of the buccal mucosa; extensive esophageal erosions with a positive Nikolsky sign	Tongue biopsy showed erosion, chronic inflammation, and suprabasal acantholysis. DIF demonstrated C3, IgG, and IgA deposition along the basement membrane zone and intercellular IgG deposition.
DiSantis et al., 2019 [28]	14/M	Lips, buccal mucosa, palate, tongue, genital and perianal regions; palms, fingertips, and axillae	One-month history of severe painful diffuse oral ulcerations, dry cracked and desquamating lips, and reduced oral intake. Oral examination revealed extensive ulceration of the buccal, palatal, and lingual mucosa covered by a purulent white film. Associated genital bullae, perianal erosions, acral desquamation, and targetoid palmar lesions were present.	Lower-lip biopsy showed suprabasal acantholysis. ELISA was positive for envoplakin antibodies, supporting PNP. DIF and IIF were negative.
Virtuoso et al., 2022 [29]	10/M	Oral and labial mucosa	Painful, erosive, bleeding oral lesions with sialorrhea, feeding difficulties, and 6 kg weight loss; no cutaneous involvement	Mucosal biopsy showed intraepithelial acantholysis; DIF revealed intercellular IgG and complement deposition; anti-desmoglein 3 antibodies were positive
Sonosaki et al., 2016 [30]	4/M	Oral mucosa, lips, tongue, and esophagus	Painful oral sores progressing to swelling and crusted erosions of the lips and tongue, resulting in inability to eat; esophageal erosions and ulcers; associated hypoalbuminemia, hypogammaglobulinemia, mild eosinophilia, and protein-losing gastroenteropathy	Suprabasal fissures and acantholytic cells in the oral mucosa; DIF showed intercellular IgG deposition in the lower epithelium; markedly elevated anti-desmoglein-3 antibodies (>1000 U/mL)
Leventhal et al., 2017 [31]	17/F	Gingiva	Eight-year history of severe diffuse gingival hypertrophy; vegetating gingival lesions covering the teeth, with overlying erythema and erosions	Suprabasal acantholysis; DIF demonstrated intercellular IgG and C3 deposition; indirect immunofluorescence showed positive intercellular IgG binding; anti-desmoglein 1 and 3 antibodies were negative, whereas anti-desmocollin 3 antibodies were positive

**Table 2:** The main demographic and clinical findings, treatment approaches, and outcomes.

Characteristic	Summary of Findings
Included studies	25 studies
Total number of patients	51 patients with documented oral/orofacial involvement *
Age range	2–17 years
Sex distribution	Male predominance; exact overall percentages could not be reliably calculated because sex was not separately reported for the 23/24 patients with oral involvement in the largest retrospective PV cohort *
General health conditions/comorbidities	Most reports did not describe major pre-existing systemic disease. Reported associated conditions included Castleman disease, eosinophilic esophagitis, protein-losing gastroenteropathy, and severe malnutrition.
Main oral clinical features	Painful oral erosions and ulcerations, vesiculobullous lesions, gingival involvement/desquamative gingivitis, stomatitis, and lip/perioral involvement. Functional consequences included feeding difficulty, dysphagia, reduced oral intake, weight loss, and malnutrition in severe cases.
Extraoral clinical features	Cutaneous involvement was common in addition to oral disease. Genital and ocular involvement was also reported, while esophageal involvement occurred in selected cases.
Main diagnostic findings	Histopathology predominantly demonstrated suprabasal acantholysis. DIF commonly showed intercellular IgG ± C3 deposition, while serological testing frequently demonstrated anti-DSG3 and/or anti-DSG1 antibodies.
Main treatments	Systemic corticosteroids were the principal treatment, frequently combined with steroid-sparing immunosuppressants, particularly azathioprine and mycophenolate mofetil. Topical corticosteroids were used in selected localized oral cases. Rituximab was mainly used for severe or refractory disease.
Clinical outcomes	Most patients demonstrated clinical improvement or remission. However, relapses were common in some cohorts, particularly during or following treatment tapering. PNP/PAMS cases generally showed more complex clinical courses and poorer outcomes.
Follow-up	Follow-up duration was heterogeneous and inconsistently reported across the included studies. In the largest PV cohort, mean follow-up was 15.9 ± 7.4 months.

* Important note: Mahajan et al. [17] reported 24 patients with PV, of whom 23 had oral mucosal involvement and one had cutaneous-limited disease. Sex was reported only for the complete PV cohort (15 males/9 females) and not separately for the 23 patients with oral involvement.

## Data Availability

The original data presented in the study are included in the article; further inquiries can be directed to the corresponding author.

## References

[1] Aleksijević L.H., Prpić J., Urek M.M., Pezelj-Ribarić S., Ivančić-Jokić N., Bukmir R.P., Aleksijević M., Glažar I. (2022). Oral Mucosal Lesions in Childhood. Dent. J..

[2] Hong C.H.L., Dean D.R., Hull K., Hu S.J., Sim Y.F., Nadeau C., Gonçalves S., Lodi G., Hodgson T.A. (2019). World Workshop on Oral Medicine VII: Relative frequency of oral mucosal lesions in children, a scoping review. Oral Dis..

[3] R H., Ramani P., Tilakaratne W.M., Sukumaran G., Ramasubramanian A., Krishnan R.P. (2022). Critical appraisal of different triggering pathways for the pathobiology of pemphigus vulgaris—A review. Oral Dis..

[4] Schmidt E., Kasperkiewicz M., Joly P. (2019). Pemphigus. Lancet.

[5] Le M., Ghazawi F.M., Miedzybrodzki B., Chauvin P.J., Jafarian F. (2019). Successful management of pediatric oral pemphigus vulgaris with topical corticosteroid monotherapy. Pediatr. Dermatol..

[6] Sriram S., Hasan S., Mansoori S., Saeed S., Banerjee A., Ramalingam K. (2024). Juvenile pemphigus vulgaris: Literature review and a rare case report. Clin. Case Rep..

[7] Sriram S., Jayakanth M., Alam T., Saeed S., Hasan S. (2025). Juvenile pemphigus vulgaris: A narrative review. Medicine.

[8] Filho G.T.L., Barbosa N.L.S., de Abreu E.M.V., da Costa K.V.T., Meneses K.C.B., Silva R.N., Ferreira S.M.S. (2021). Childhood pemphigus vulgaris is a challenging diagnosis. Autops. Case Rep..

[9] Surya V., Kumar P., Gupta S., Urs A. (2018). Childhood pemphigus vulgaris: Report of two cases with emphasis on diagnostic approach. Contemp. Clin. Dent..

[10] Chen I.H., Mu S.C., Tsai D., Chou Y.Y., Wang L.F., Wang L.J. (2016). Oral Ulcers as an Initial Presentation of Juvenile Pemphigus: A Case Report. Pediatr. Neonatol..

[11] Gue S., Huang G., Moore L., Hammond P., Boros C.A. (2017). Pemphigus Vulgaris and Eosinophilic Esophagitis in a 13-Year-Old Boy: Case Report and Review of the Literature. Pediatr. Dermatol..

[12] Suwarsa O., Sutedja E., Dharmadji H.P., Kusuma P., Rahardja J., Hindritiani R., Gunawan H. (2017). The Rare Case of Pemphigus Vegetans in Association with Malnutrition Children in the Multidisciplinary Management. Case Rep. Dermatol..

[13] Bajpai M., Pardhe N., Chandolia B. (2016). Lichenoid Reaction—A Diagnostic Dilemma. http://www.jamc.ayubmed.edu.pk636.

[14] Han S.P., Fu L.S., Chen L.J. (2019). Masked pemphigus among pediatric patients with Castleman’s disease. Int. J. Rheum. Dis..

[15] Lamboglia A.L.C., Gubitosi A.M., Bakerdjian C.G., Larraz G.G. (2018). Pemphigus vulgaris in pediatrics: A case report. Rev. Chil. Pediatr..

[16] Sathishkumar D., Agrawal P., Baitule A.M., Thomas M., Eapen A., Kumar S., George R. (2021). Paraneoplastic autoimmune multiorgan syndrome: A retrospective study from a tertiary care center in South India. Indian Dermatol. Online J..

[17] Mahajan R., Handa S., Kumar S., Chatterji D., Saikia U.N., De D. (2022). Pediatric autoimmune blistering disorders—A five-year demographic profile and therapy experience. Int. J. Dermatol..

[18] Barry K.K., Plumptre I., Bazewicz C.G., Green B.P., Treat J.R., Hughes M., Morel K.D., Wiss K., Liang M.G. (2023). Paraneoplastic pemphigus associated with Castleman disease: A multicenter case series. Pediatr. Dermatol..

[19] Vezzoli P., Parietti M., Carugno A., Di Mercurio M., Benaglia C., Zussino M., Cavalli R., Sena P., Berti E. (2022). Childhood Pemphigus Vulgaris during COVID-19 Outbreak Successfully Treated with Prednisone and Azathioprine: A Case Report and Literature Review. J. Clin. Med..

[20] Irrera M., Bozzola E., Cardoni A., DeVito R., Diociaiuti A., El Hachem M., Girardi K., Marchesi A., Villani A. (2023). Paraneoplastic pemphigus and Castleman’s disease: A case report and a revision of the literature. Ital. J. Pediatr..

[21] Akham R., Bhatia R., Paonam R., Hazarika N. (2023). Rescue treatment with intravenous immunoglobulin and amniotic membrane dressing in refractory paediatric pemphigus vulgaris with sepsis. BMJ Case Rep..

[22] Yun S., Jenkins B.A., Scollan M.E., Defelice A.R., Leiferman K.M., Milner J.D., Lauren C.T. (2025). Pemphigus Vulgaris with Esophageal Involvement in an Atopic Child Successfully Treated with Dupilumab. Pediatr. Dermatol..

[23] Zhang R., Liu J., Nie N., Wang D., Wu J., Zhang H., Zhang R., Gao S., Bai C., Lin Y. (2024). Case Report: A rare pediatric case of paraneoplastic pemphigus associated with Castleman disease misdiagnosed as Behçets disease. Front. Pediatr..

[24] Belgaumkar V.A., Raj R., Pradhan S., Nile S., Bhale G. (2025). Childhood vesiculobullous eruptions: A case report of a rare conundrum. Iran. J. Dermatol..

[25] Govindarajulu S.M., Nair A., Reddy P.K.S., Sumathy T.K. (2025). Scrotal Tongue in Juvenile Oral Pemphigus Vulgaris. Clin. Dermatol. Rev..

[26] De Felici Del Giudice M.B., Calanca C., Sassetti C., Caffarelli C., Feliciani C., Esposito S. (2023). Severe Pemphigus Vulgaris Resistant to Conventional Therapies and with Hypersensitivity to Rituximab in a 12-Year-Old Child. Children.

[27] Fidder S.A.R., Bolling M.C., Diercks G.F.H., Pas H.H., Hooimeijer L.H.L., Bungener L.B., Willemse B.W.M., Scheenstra R., Stapelbroek J.M., van der Doef H.P.J. (2021). Paraneoplastic pemphigus associated with post-transplant lymphoproliferative disorder after small bowel transplantation. Pediatr. Transplant..

[28] DiSantis F., Hames N., Weiss L. (2019). A Teenager with Painful Oral and Genital Lesions. Clin. Pediatr..

[29] Virtuoso J., Ribeiro J.F., Silva Í.S., Santos S., Fernandes P., Cabral F. (2022). Pemphigus vulgaris: A rare disease in childhood. J. Paediatr. Child Health.

[30] Sonosaki T., Miyagi T., Yamaguchi S., Arakaki O., Yamamoto Y., Arakaki M., Nakamura S., Kinjo N., Uezato H., Takahashi K. (2016). Pediatric case of oral mucous pemphigus complicated by protein-losing gastroenteropathy. J. Dermatol..

[31] Leventhal J.S., Haberland C.M., Cowper S.E., Tomayko M.M. (2017). Image Gallery: Juvenile autoimmune pemphigus presenting with diffuse gingival hypertrophy and antibodies against desmocollin 3. Br. J. Dermatol..

[32] Mabrouk D., Ahmed A.R. (2011). Analysis of Current Therapy and Clinical Outcome in Childhood Pemphigus Vulgaris. Pediatr. Dermatol..

[33] Eskiocak A.H., Ozkesici B., Uzun S. (2016). Familial Pemphigus Vulgaris Occured in a Father and Son as the First Confirmed Cases. Case Rep. Dermatol. Med..

[34] Hans-Filho G., Aoki V., Bittner N.R.H., Bittner G.C. (2018). Fogo selvagem: Endemic pemphigus foliaceus. An. Bras. Dermatol..

[35] Lim Y.L., Bohelay G., Hanakawa S., Musette P., Janela B. (2022). Autoimmune Pemphigus: Latest Advances and Emerging Therapies. Front. Mol. Biosci..

[36] Drerup K., Fölster-Holst R. (2020). Neonatal pemphigus vulgaris. JDDG J. Dtsch. Dermatol. Ges..

[37] Rosi-Schumacher M., Baker J., Waris J., Seiffert-Sinha K., Sinha A.A. (2023). Worldwide epidemiologic factors in pemphigus vulgaris and bullous pemphigoid. Front. Immunol..

[38] Rodriguez Baisi K., Wentworth A., Chattha A.J., DiCaudo D.J., Mangold A., Nelson S.A., Siegfried E., Wieland C.N., Tollefson M. (2021). A rare case of childhood mucous membrane pemphigoid involving the oral and genital mucosa. Pediatr. Dermatol..

[39] Khodeir J., Ohanian P., Saleh S., Karam K., Dib E., Chebbo H., Fiani E. (2025). Rare Coexistence of Pemphigus Vulgaris and Eosinophilic Esophagitis: A Report of Two Cases. Clin. Case Rep..

[40] Ishige T., Kaneko H., Suzuki T., Miyazawa R., Tomomasa T., Kubota M., Morikawa A. (2008). Pemphigus vulgaris as a possible cause of protein-losing gastroenteropathy: A case report. J. Paediatr. Child Health.

[41] Moro F., Sinagra J.L.M., Salemme A., Fania L., Mariotti F., Pira A., Didona B., Di Zenzo G. (2023). Pemphigus: Trigger and predisposing factors. Front. Med..

[42] Horiguchi Y., Fujii S., Takase S., Mori R. (2005). Case of pemphigus vulgaris in oral mucosa showing an extension to the body surface with recurrent varicella. Int. J. Dermatol..

[43] Hertl M., Jedlickova H., Karpati S., Marinovic B., Uzun S., Yayli S., Mimouni D., Borradori L., Feliciani C., Ioannides D. (2015). Pemphigus. S2 Guideline for diagnosis and treatment—Guided by the European Dermatology Forum (EDF) in cooperation with the European Academy of Dermatology and Venereology (EADV). J. Eur. Acad. Dermatol. Venereol..

[44] Malik A.M., Tupchong S., Huang S., Are A., Hsu S., Motaparthi K. (2021). An Updated Review of Pemphigus Diseases. Medicina.

